# Long noncoding and micro-RNA expression in a model of articular chondrocyte degeneration induced by stromal cell-derived factor-1

**DOI:** 10.2478/abm-2022-0021

**Published:** 2022-08-31

**Authors:** Guoliang Wang, Lu He, Yaoyu Xiang, Di Jia, Yanlin Li

**Affiliations:** Department of Sports Medicine, First Affiliated Hospital of Kunming Medical University, Kunming, Yunnan 650032, China; Kunming Medical University, Kunming, Yunnan 650032, China

**Keywords:** chondrocytes, high-throughput nucleotide sequencing, microRNAs, osteoarthritis, RNA, long noncoding

## Abstract

**Background:**

Gene regulatory network analysis has found that long noncoding ribonucleic acids (lncRNAs) are strongly associated with the pathogenesis of osteoarthritis.

**Objectives:**

To determine the differential expression of lncRNAs and microRNAs (miRNAs) in normal chondrocytes and those from a model of articular chondrocyte degeneration.

**Methods:**

Chondrocytes were cultured from cartilage obtained from patients diagnosed with osteoarthritis of the knee. Stromal cell-derived factor-1 (SDF-1) was used to induce their degeneration. Total RNA was extracted, analyzed, amplified, labeled, and hybridized on a chip to determine expression. The set of enriched differentially expressed miRNAs was analyzed by gene ontology and the Kyoto Encyclopedia of Genes and Genomes to describe the functional properties of the key biological processes and pathways. We conducted a bioinformatics analysis using Cytoscape to elucidate the interactions between miRNAs and proteins.

**Results:**

We found that the expression of 186 lncRNAs was significantly different in the model of chondrocyte degeneration, in which 88 lncRNAs were upregulated, and 98 were downregulated. Expression of 684 miRNAs was significantly different. Analysis of the protein–protein interaction (PPI) network indicated that the genes for CXCL10, ISG15, MYC, MX1, OASL, IFIT1, RSAD2, MX2, IFI44L, and BST2 are the top 10 core genes, identifying the most important functional modules to elucidate the differential expression of miRNAs.

**Conclusions:**

These data may provide new insights into the molecular mechanisms of chondrocyte degeneration in osteoarthritis, and the identification of lncRNAs and miRNAs may provide potential targets for the differential diagnosis and therapy of osteoarthritis.

Osteoarthritis is a chronic and progressive multifactorial disease characterized by subchondral bone destruction, reduced numbers of chondrocytes, and degradation of the cartilaginous matrix [1,2,3]. Long noncoding ribonucleic acids (lncRNAs) and microRNAs (miRNAs) play important roles in mediating gene regulatory pathways in the pathogenesis of osteoarthritis and other diseases, including acute early phase spinal cord injury [4,5,6]. Aberrant expression of lncRNAs and miRNAs is associated with the development of osteoarthritis and may play regulatory roles in its pathogenesis [7,8,9]. About 4700 lncRNAs are expressed aberrantly in cartilage from patients with osteoarthritis compared with normal cartilage from control patients [10]. lncRNAs play an important regulatory role in the processes of joint synovial inflammation, cartilage matrix synthesis and metabolism, angiogenesis, chondrocyte autophagy, apoptosis, and other factors associated with osteoarthritis [11,12,13].

Stromal cell-derived factor-1 (SDF-1) is found at significantly higher levels in the synovial fluid of patients with osteoarthritis and has strong effects to induce cartilage matrix degradation. The SDF-1/chemokine (CXC motif) receptor 4 (CXCR4) signaling pathway plays a key role in the pathological process of cartilage degeneration in animal models and increases interleukin (IL)-6 production by human synovial fibroblasts [14,15,16,17]. Synovial tissue of the knee joints in patients with osteoarthritis can produce SDF-1 at a higher concentration than the synovial tissue of healthy knee joints. SDF-1 can interact with CXCR4-specific receptors on the surface of cartilage to activate the SDF-1/CXCR4 signaling pathway, which activates the extracellular signal-regulating enzyme (Erk) and related kinase (p38 mitogen-activated protein (MAP) kinase) signaling pathways, promoting the release of matrix metalloproteinases (MMP) from the cartilage matrix, which degrade the type II collagen and aggrecan substrates in the cartilage matrix, ultimately degenerating the articular cartilage and inducing osteoarthritis [18,19,20]. lncRNA-H19 stimulates osteogenic differentiation of bone marrow mesenchymal stem cells by regulating SDP-1 expression via miRNA-149 [21]. miRNA-126-silenced mice showed that miRNA-126 can regulate the expression of SDF-1 in endothelial cells [22]. miRNA-141-3p regulator of SDF-1 in bone marrow stromal cells may play an important role in the age-dependent pathophysiology of the murine and human bone marrow niche [23]. These studies indicate that the expression of SDP-1 in tissues and cells may be regulated by a multifaceted network of lncRNA and miRNA.

Here, we examined the expression of miRNAs and lncRNAs in an SDF-1-induced model of chondrocyte degeneration. Subsequently, we used microarrays to analyze the differential expression of the identified miRNAs and lncRNAs. We also analyzed the differential expression of lncRNAs in terms of transcript length distribution, classification, and exon number. Bioinformatics analysis was used to clarify the interaction between differentially expressed lncRNAs and miRNAs. A gene ontology analysis and Kyoto Encyclopedia of Genes and Genomes (KEGG) enrichment analysis were performed to identify the critical biological processes and pathways.

## Methods

### Materials and osteoarthritis cartilage modeling

All cartilage tissue was obtained from patients diagnosed with knee osteoarthritis who underwent total knee replacement at the First Affiliated Hospital of Kunming Medical University (March 2018 to March 2019). The cartilaginous tissue remaining on the surface of the tibial plateau and femoral condyle after osteotomy was collected during the surgery. The cartilage tissue specimen donors were informed of the research in writing and provided their documented consent before the specimens were collected. The present study was approved by the ethics committee of the First Affiliated Hospital of Kunming Medical University (2018 Lun Shen L No. 21), and the study protocols were in compliance with relevant national regulations and laws, including the ethical principles of the China Food and Drug Administration Good Clinical Practice for Medical Devices and People's Republic of China Regulations for the Management of Medical Institutions (promulgated by the Order No. 149 of the State Council on February 26, 1994; and revised in accordance with the Decision of the State Council on Amending Some Administrative Regulations on February 6, 2016), and international medical ethics documents, including the International Conference on Harmonisation Good Clinical Practice (ICH-GCP) and the Declaration of Helsinki and its contemporary (2013) revisions. After a diagnosis of osteoarthritis in accordance with the criteria described by Altman et al. [24], 10 patients (4 male and 6 female) underwent artificial knee arthroplasty due to osteoarthritis. The patients were aged from 55 to 75 years and had a gross visual grade of cartilage degeneration score of 0 or 1 point, where 0 points indicated a smooth articular surface and usual color, and 1 point indicated a rough articular surface, small cracks, and dark color [25]. Patients with liver or kidney disease, connective tissue disease, endocrine disease, serious cardiovascular disease, and tumors were excluded. The cartilage tissue was trimmed to dimensions 2 mm × 2 mm × 1 mm under aseptic conditions. Ten pieces (100 pieces in total from the 10 different patients) of cartilage tissue were placed separately into preprepared high-glucose Dulbecco's modified Eagle's medium (DMEM) for digestion and culture. Chondrocytes from the first-generation culture were divided equally without special selection into an experimental and control group (n = 3 each). The density of autologous chondrocytes in the carrier was about 1.6–2.0 × 10^5^ cells/cm^2^. The cell culture medium in the 2 groups was high-glucose DMEM containing 10% fetal bovine serum and penicillin–streptomycin. In the experimental group, 100 ng/mL SDF-1 (R&D Systems) was added to the chondrocytes [17], and the control group was untreated. The chondrocytes in the 2 groups were cultured under the same conditions for 48 h using an improved method [19, 20], although a vehicle control was not used to conserve resources.

### RNA extraction

Total RNA samples were extracted using an RNeasy Mini Kit (catalog No. 74106; Qiagen). The extraction was performed in accordance with the standard operating procedure handbook provided by the manufacturer. The extracted total RNA was examined qualitatively using a Bioanalyzer 2100 system (Agilent Technologies) and quantified using a Qubit 3.0 Fluorometer (Life Technologies) and NanoDrop One spectrophotometer (Thermo Fisher Scientific).

### RNA amplification and labeling

Total RNA was amplified and labeled using a Low-Input QuickAmp WT Labeling Kit (catalog No. 5190-2943; Agilent Technologies) according to the kit instructions; the labeled complementary RNA was purified using an RNeasy Mini Kit (catalog No. 74106; Qiagen).

### Hybridization

The NimbleGen SeqCap EZ Hybridization and Wash Kit (Roche) for permutation hybridization was used to enrich specific target regions according to protocols specified by the manufacturer.

### Data collection

An Axon 4000B fluorescent scanner (Molecular Probes) was used to scan hybridized microarray slides and convert the scanning signal into a digital signal, and the low quality and weak signal data points were excluded. Fold change (FC) ≥2 was the cutoff criterion. A Student *t* test was performed to calculate the scanning signal values of the 2 groups and to obtain the log_2_ (ratio) and *P* value for each probe. When the ratio of the intensity of the hybridization signal between the experimental group and the control group was ≥2, the expression was defined as upregulated; otherwise, the expression was defined as downregulated. The miRNA screening conditions were FC ≥2 and *P* < 0.05 or log_2_ (ratio) ≥0.8.

### Gene Ontology and KEGG analysis

Gene ontology (GO) analysis was performed to describe the functional properties of differentially expressed miRNAs. GO analysis included molecular function (MF), biological process (BP), and cellular component (CC). KEGG signaling pathway analysis was performed to describe the biological pathways of differentially expressed miRNAs.

### Protein–protein interaction network analysis

To elucidate the interactions between differentially expressed miRNAs, a database of interacting genes was searched, and Cytoscape visualization was used to integrate biological models with biological graphics visualization tools for molecular interaction networks [26]. Differentially expressed miRNAs with FC >4 and *P* < 0.05 were identified, and the STRING online tool [27] was used to analyze differentially expressed miRNAs with a combined protein–protein interaction (PPI) score >0.4 as the cutoff value.

### Statistical analysis

Data were analyzed using SPSS Statistics for Windows (version 17.0; SPSS). Differentially expressed (DE) levels of miRNAs and lncRNAs were compared using paired-sample *t* tests. Student *t* tests were to compare values between the groups. The differentially expressed lncRNAs and differentially expressed miRNAs with a FC threshold >2 and *P* < 0.05 were regarded as significant.

## Results

### Morphological changes in cell culture

**Figure 1:**
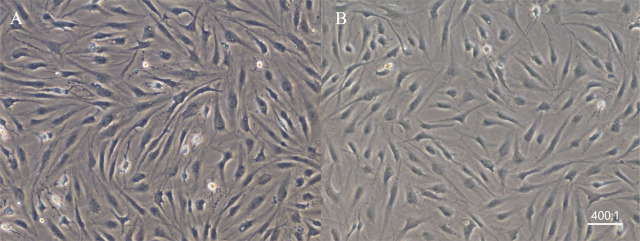
Morphology of chondrocytes in the 2 groups (×100). **A.** Chondrocytes in the experimental group were cultured with SDF-1 for 48 h. **B.** Morphology of chondrocytes in the control group. SDF-1, stromal cell-derived factor-1.

Chondrocytes from the first-generation culture of osteoarthritis tissue were cultured with SDF-1 for 48 h. The chondrocytes in the experimental group were irregular and long spindle–shaped, with a low refractive index and fuzzy structure in living cells, while the chondrocytes in the control group were spindle-shaped or oval, with an intact nucleus, high refractive index, and clear structure in living cells (**Figures 1A and B**).

### Difference visualization

**Figure 2:**
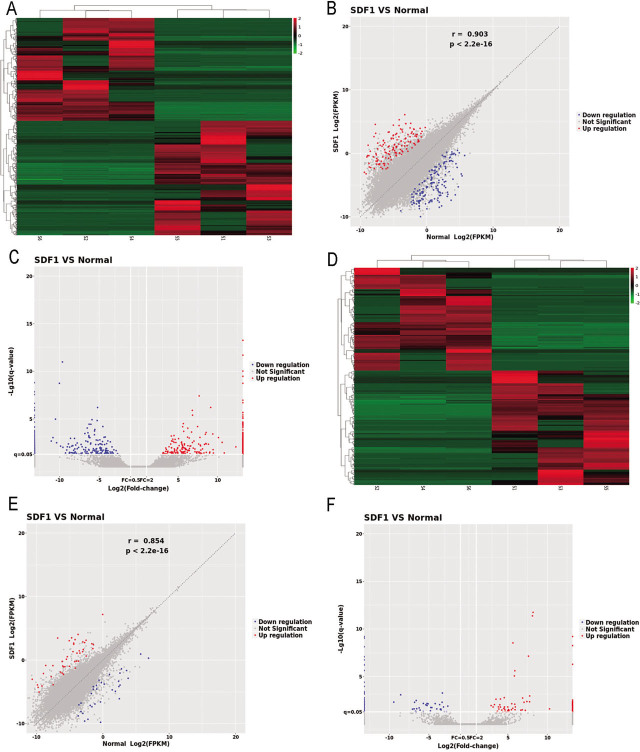
Analysis of miRNAs and lncRNAs. **A.** Heatmap of the differentially expressed (DE) miRNAs. **B.** Scatter plot of the DE miRNAs. **C.** Volcano plot of the DE miRNAs. **D.** Heatmap of the DE lncRNAs. **E.** Scatter plot of the DE lncRNAs. **F.** Volcano plot of the DE lncRNAs. In the heatmap, red represents upregulated miRNAs or lncRNAs, and green represents downregulated miRNAs or lncRNAs. In the scatter plot, the X and Y values are the average normalized signal values, shown on a log_2_ scale. The red and green lines were set as FC lines with a default change of 2.0. Red points (FC >2) indicate upregulated miRNAs or lncRNAs, and blue points (FC ≤2) indicate downregulated miRNAs or lncRNAs. In the volcano plot, the X-axis is the FC (log_2_), and the Y-axis is P (−log_10_). Red points (FC >2) indicate upregulated miRNAs or lncRNAs, and blue points (FC ≤2) indicate downregulated miRNAs or lncRNAs. DE, differentially expressed; FC, fold change; FPKM, fragments per kilobase of transcript per million mapped fragments; lncRNAs, long noncoding ribonucleic acids; miRNAs, microRNAs; SDF1, stromal cell-derived factor-1 treated articular chondrocytes. Normal indicates articular chondrocytes untreated with SDF-1.

lncRNA analysis revealed a total of 52,741 lncRNAs with changed expression. Further analysis showed that of these, the expression of 186 lncRNAs was changed significantly; 88 were upregulated, and 98 were downregulated. A total of 119,205 miRNAs had changed their level of expression, and the expression of 684 miRNAs had changed significantly. The heatmap, scatter plot, and volcano plot of the differentially expressed miRNAs and lncRNAs are shown in **Figure 2** (miRNAs, **A–C**; lncRNAs, **D–F**).

### Differential expression analysis of lncRNAs

**Figure 3:**
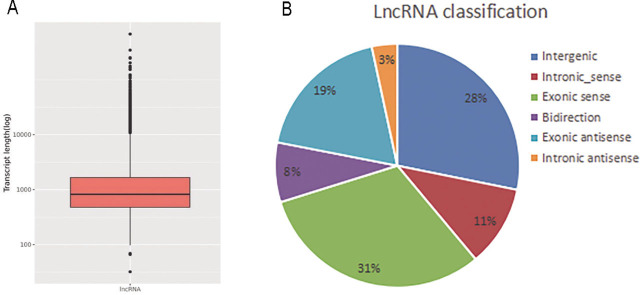
Expression signatures of dysregulated lncRNAs in SDF-1-induced articular chondrocyte degeneration. **A.** Length distribution showed that dysregulated lncRNAs were mainly concentrated between 700 bp and 3000 bp. **B.** Differential lncRNAs were classified according to their genomic architecture.

The top 10 most upregulated and most downregulated lncRNAs in the experimental group are shown in **Table 1**. Horizontal comparisons based on the transcript structure of the lncRNAs were performed, including the transcript length distribution, classification, and exon quantity differences. The length of lncRNAs was mainly concentrated at approximately 1000 bp, and lncRNAs constituted various RNA molecules (**Figure 3A**). The traditional classification method of lncRNAs is based on the location of the transcript in the genome and includes 5 major categories: (1) the sense group, (2) the antisense group, (3) the bidirectional group, (4) the intronic group, and (5) the intergenic group (**Figure 3B**).

**Table 1:** Top 10 most upregulated and most downregulated lncRNAs in chondrocytes from the SDF-1-induced model of articular chondrocyte degeneration

**Upregulated lncRNAs**			**Downregulated lncRNAs**		

**lncRNA ID**	***P***	**FC**	**lncRNA ID**	***P***	**FC**
NONHSAT094312.2	5.51E–05	10.27	NONHSAT246243.1	4.01E–06	9.44
NONHSAT060379.2	3.42E–17	8.19	NONHSAT217441.1	3.97E–07	8.57
NONHSAT207507.1	1.60E–16	8.12	NONHSAT238505.1	6.22E–05	7.07
NONHSAT166467.1	5.44E–07	7.77	NONHSAT258030.1	5.57E–05	6.86
NONHSAT198879.1	3.39E–06	7.75	NONHSAT176410.1	4.59E–06	6.81
NONHSAT248596.1	1.21E–11	7.61	NONHSAT022132.2	0.000138	6.68
NONHSAT152279.1	2.89E–06	7.41	NONHSAT119402.2	2.11E–06	6.59
NONHSAT000091.2	9.96E–05	7.21	NONHSAT022138.2	0.000169	6.58
ENST00000559458	4.66E–06	7.05	NONHSAT229871.1	5.25E–05	6.53
NONHSAT038052.2	1.00E–06	6.87	NONHSAT225394.1	4.07E–05	6.23

FC, fold change; SDF-1, stromal cell-derived factor-1.

### Gene ontology and KEGG analyses

**Figure 4:**
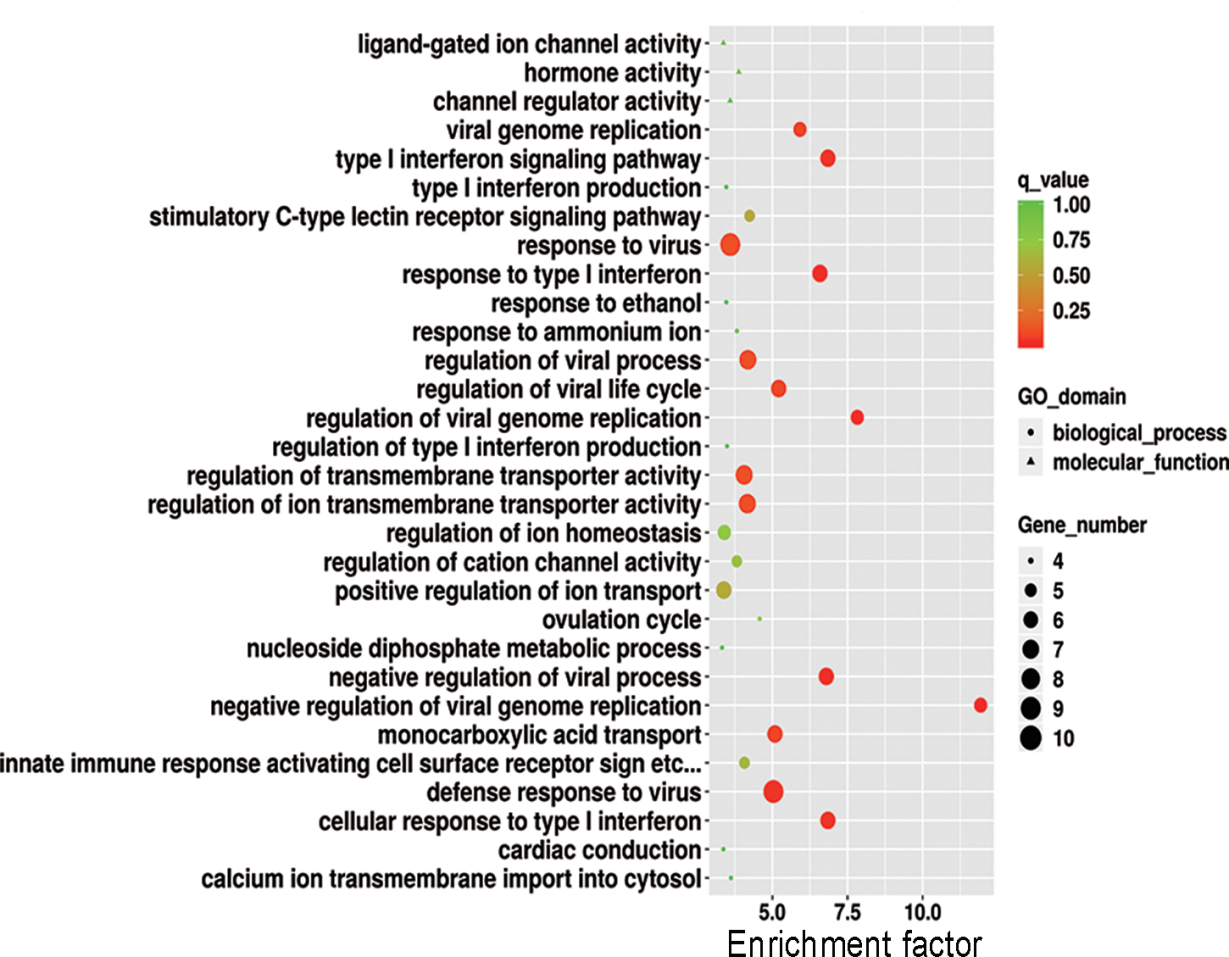
GO analysis of differentially expressed genes. GO annotations of mRNAs with top 30 enrichment scores. The circles represent biological processes; the triangles represent cell components; and the squares represent MF. GO, gene ontogeny; MF, molecular functions.

**Figure 5:**
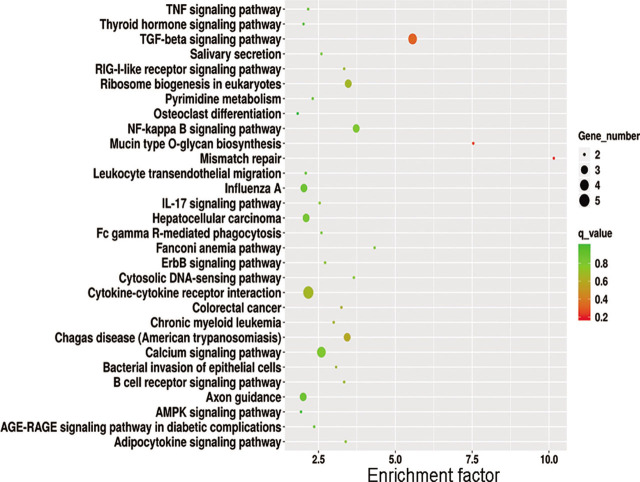
KEGG signaling pathway analysis of differentially expressed genes. Top 30 for KEGG enrichment. KEGG, Kyoto Encyclopedia of Genes and Genomes.

The GO analysis showed that the signaling pathways of miRNAs and their target genes are enriched in receptor regulation activities (MF), secondary lysosomes (cell components), lipopolysaccharide regulatory signaling pathways (biological processes), type I interferon signaling pathways, and ionic transmembrane transporter activity regulation (**Figure 4**). Pathway analysis indicated that miRNAs and their target genes are enriched in cytokine–cytokine receptor interactions, osteoclast differentiation, the nuclear factor κ-light-chain-enhancer of activated B cells (NF-κB) signaling pathway, the transforming growth factor (TGF-β) signaling pathway, and the ion signaling pathway, as shown in **Figure 5**.

### PPI network construction

**Figure 6:**
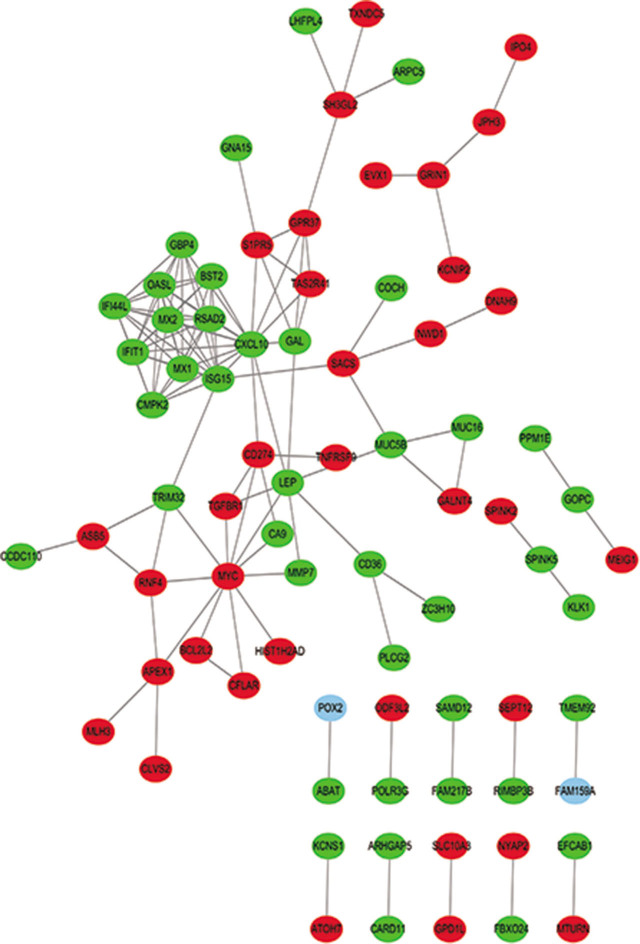
PPI network analysis of the top 10 differentially expressed genes. Nodes represent genes for the proteins indicated. Lines indicate interactions between genes. Red indicates upregulated genes, and green indicates downregulated genes. PPI, protein–protein interaction.

The PPI network identified genes for 10 proteins, interferon-γ (IFN-γ) inducible CXC 10 kDa chemokine chemotactic for monocytes and T-lymphocytes (CXCL10), interferon-α-stimulated gene 15-kDa protein (ISG15), v-myc myelocytomatosis viral oncogene homolog protein (MYC), interferon inducible myxovirus resistance protein MxA p78 (MX1), p59 2′,5′-oligoadenylate synthetase-like protein (OASL), interferon-induced with tetratricopeptide repeats 1 protein (IFIT1), radical S-adenosyl methionine domain containing 2 protein (RSAD2), second interferon-induced myxo-virus resistance 2 protein MxB p78 (MX2), interferon-induced protein 44 like protein (IFI44L), and bone marrow stromal tetherin antigen 2 (BST2), that have a higher possibility of being involved in the mechanism of chondrocyte degeneration (**Table 2**) and showed a network of upregulated and downregulated genes (**Figure 6**).

**Table 2:** PPI network core (top 10) genes in the SDF-1-induced model of articular chondrocyte degeneration

**Protein**	**Degree**	**Eccentricity**	**Edge count**
CXCL10	16	4	16
ISG15	12	4	12
MYC	11	5	11
MX1	10	5	10
OASL	10	5	10
IFIT1	10	5	10
RSAD2	10	5	10
MX2	10	5	10
IFI44L	10	5	10
BST2	8	5	8

BST2, bone marrow stromal tetherin antigen 2; CXCL10, interferon γ inducible CXC 10 kDa chemokine chemotactic for monocytes and T-lymphocytes; IFI44L, interferon-induced protein 44 like protein; IFIT1, interferon-induced with tetratricopeptide repeats 1 protein; ISG15, interferon-α-stimulated gene 15-kDa protein; MX1, interferon inducible myxovirus resistance protein MxA p78; MX2, second interferon-induced myxovirus resistance 2 protein MxB p78; MYC, v-myc myelocytomatosis viral oncogene homolog protein; OASL, p59 2′,5′-oligoadenylate synthetase-like protein; PPI, protein–protein interaction; RSAD2, radical S-adenosyl methionine domain containing 2 protein; SDF-1, stromal cell-derived factor-1.

## Discussion

RNA expression data have been uploaded to the Sequence Read Archive database, and the BioProject ID is PRJNA638147. lncRNAs can compete with competing endogenous RNAs (ceRNA), as miRNA sponges, to play a regulatory role [7]. lncRNAs participate in gene regulation as guides, signals, baits, and scaffolds. The specific regulatory mechanism is mainly divided into 4 aspects, which regulate the degeneration of articular cartilage by regulating transcription factors and transcription processes and mediating post-transcriptional regulation of miRNA and mRNA, and regulation of nuclear structure [28]. Most research studies have focused on the function of lncRNAs as a “sponge,” in which lncRNA undergoes an endogenous competition interaction with miRNA that has a shared binding site to inhibit the regulatory effect of the miRNA on target mRNA, thereby affecting protein expression. The more binding sites there are, the stronger the “sponge effect” and the more obvious the inhibitory effect of lncRNA on miRNA. Under normal circumstances, various RNAs (such as lncRNA, miRNA, and mRNA) maintain a balanced state, and when an RNA is abnormally expressed, the balance is disrupted, and this results in disease [29]. Noncoding RNA with a common response element (miRNA response elements [MRE]) can compete with mRNA endogenously to bind miRNA and inhibit miRNA-mediated negative regulation of mRNA. Similarly, reducing ceRNA levels upregulate target gene expression, which may ultimately affect cellular biological processes [30].

Receptor regulatory factors such as Toll-like receptors (TLRs) are evolutionarily conserved molecules that promote immune responses by recognizing molecular patterns related to microorganisms. During infection, TLR signaling is necessary for the proper activation of the immune response [31]. TLRs produce large amounts of interleukin (IL)-1β and tumor necrosis factor (TNF)-α inflammatory factors by activating the NK-κB inflammatory signaling pathway. Liu et al. [32] found that the expression of TLR-2, NF-κB, MMP-13, and related inflammatory factors was significantly upregulated with the severity of osteoarthritis lesions, suggesting that the TLR-2/NF-κB signaling pathway may be involved in the occurrence of osteoarthritis.

When intra-articular hemorrhage occurs, lysosomes release degrading enzymes, and decreased proteoglycan concentration reduces chondrocyte synthesis activity and induces articular cartilage degeneration [33]. A high concentration of SDF-1 can increase its interaction with CXCR4 on the surface of chondrocytes and accelerate the degradation of type II collagen through the upregulation of MMPs, also leading to cartilage degeneration [34]. Chondrocytes are nonexcitable cells. However, the multiple ion channels present on the cell membrane are the basis for the cell to carry out various life activities, including transporting ions necessary for cell metabolism, regulating osmotic pressure inside and outside the cell, participating in the formation of electrical impulses, and mediating in signal transmission to adapt organisms to environmental conditions [35, 36].

Pathway analysis showed that miRNAs and their target genes were enriched in cytokine–cytokine receptor interaction, osteoclast differentiation, NF-κB signaling pathway, TGF-β signaling pathway, and Ca^2+^ signaling. Cytokines regulate the balance of anabolic and catabolic metabolism of cartilage matrix. They are divided into catabolic cytokines and anabolic cytokines according to their roles in the regulation of metabolism. The balance and imbalance between them are root causes of the degradation and destruction of the cartilage matrix in osteoarthritis. Cytokines, including TNF-α, IL-1, IL-6, IL-2, and IFN-γ, are involved in this pathway. These cytokines penetrate the synovium to induce an inflammatory response. In addition, they can activate synovial cells and stimulate the release of MMPs into the synovial fluid, leading to cartilage degradation [37, 38].

Currently, the most studied cytokines that promote chondrocyte catabolism are IL-1 and TNF-α. IL-1 not only inhibits the synthesis of the characteristic matrix components type II collagen and aggrecan by articular chondrocytes, but also stimulates articular chondrocytes to secrete protease that degrades cartilage matrix components, inhibits the expression of type I and type II collagen by articular chondrocytes, and promotes the degeneration of articular chondrocytes [39]. TNF-α also plays an important role in osteoarthritis cartilage degeneration. The mechanism of action of TNF-α is similar to that of IL-1 and includes promoting the generation of MMP and inhibiting the synthesis of cartilage matrix. TNF inhibits the expression of type II collagen and connexin genes through the MAP kinase/extracellular signal-regulated kinase (ERK) kinase (MEK) 1/2 and NF-κB pathways, which in turn interferes with the synthesis and reconstruction of articular cartilage [40]. The NF-κB transcription factor regulates gene expression, and the NF-κB signaling pathway is activated in articular cartilage and synovial cells in osteoarthritis [41]. NF-κB regulates the response to joint injury and inflammation by regulating cytokines, including IL-1β and TNF-α [39,40,41,42,43,44,45,46].

The PPI network revealed genes for 10 proteins that have a high possibility of being associated with the pathological process of chondrocyte degeneration: CXCL10, ISG15, MYC, MX1, OASL, IFIT1, RSAD2, MX2, IFI44L, and BST2. Chemokines, mainly CXC and CC, and their corresponding receptors are expressed in human chondrocytes, and their expression is increased in osteoarthritis articular cartilage [47]. Chemokines are involved in cartilage destruction by inducing the expression of related enzymes, mainly *N*-acetyl-β-d-glucosidase (NAG) and MMP. NAG is the main lysosomal glycosidase in osteoarthritis synovial fluid and catalyzes the hydrolysis of glucosamine polysaccharides, causing cartilage destruction [48].

In osteoarthritis, the cartilage surface is activated by a variety of chemokines, releasing enzymes that mediate the destruction of the cartilage matrix [48, 49]. Kostopoulou et al. [50] and Tardif et al. [51] found that an osteoarthritis-related miRNA can inhibit MMP-13.

MYC is not strongly expressed in normal chondrocyte nuclei, but is scattered in apoptotic chondrocyte nuclei. The degree of articular chondrocyte apoptosis in osteoarthritis is positively correlated with the degree of cartilage degeneration, and MYC participates in the process of chondrocyte apoptosis. The mechanism of MYC causing apoptosis may be due to an imbalance in the normal cell cycle, which inhibits cell growth [52].

In-depth studies of cytokine interactions, osteoarthritis signaling pathways, and miRNAs related to lncRNAs are required to investigate the relationship between lncRNAs and miRNAs, to elucidate the molecular mechanism of osteochondrocyte degeneration, and provide a new basis and targets for the effective diagnosis and treatment of osteoarthritis.
